# Treatment with caspase-1 inhibitor diminishes kidney disease in MRL-Faslpr mice and delays systemic illness

**DOI:** 10.1136/rmdopen-2025-006194

**Published:** 2025-12-23

**Authors:** Paul Claßen, Myriam Meineck, Matthias Plath, Sabrina Saurin, Federica Fasola, Andrea Pautz, Andreas Schwarting, Simone Cosima Boedecker-Lips, Julia Weinmann-Menke

**Affiliations:** 1 Department of Internal Medicine I, University Medical Center of the Johannes Gutenberg University Mainz, Mainz, Germany; 2 Institute of Pharmacology, University Medical Center of the Johannes Gutenberg University Mainz, Mainz, Germany; 3 Acura Rheumatology Center Rhineland-Palatinate, Bad-Kreuznach, Germany; 4 Research Center for Immunotherapy (FZI), University Medical Center of the Johannes Gutenberg University Mainz, Mainz, RP, Germany

**Keywords:** Systemic Lupus Erythematosus, Lupus Nephritis, Inflammation, Autoimmune Diseases

## Abstract

**Objectives:**

To investigate the therapeutic potential of caspase-1 inhibition in systemic lupus erythematosus (SLE) and lupus nephritis (LN) using the MRL-*Fas^lpr^* mouse model.

**Methods:**

Female Murphy Roths large (MRL)-*Fas^lpr^* mice were treated with a caspase-1 inhibitor (pralnacase) or sham treatment from 2.5 to 5.5 months of age. Disease progression was assessed through analysis of systemic and renal parameters, including proteinuria, blood urea nitrogen, kidney histopathology and immune cell infiltration. Cytokine expression and caspase activity were measured to elucidate the mechanism of action. Additional experiments with interleukin (IL)-1 receptor antagonist and pancaspase inhibition treatment as well as post-disease onset intervention were conducted.

**Results:**

Caspase-1 inhibition significantly reduced systemic inflammation, lymphadenopathy and skin lesions in MRL-*Fas^lpr^* mice. Treated mice exhibited decreased proteinuria, improved renal function and reduced kidney pathology. The treatment specifically targeted caspase-1 activity, leading to decreased IL-18 levels as well as attenuated immune cell activation and infiltration in the kidneys. Selective caspase-1 inhibition showed comparable results with pancaspase inhibition. IL-1 receptor antagonist treatment did not significantly affect disease progression, suggesting IL-18 as the primary driver of pathology. Post-disease onset intervention with caspase-1 inhibition also showed efficacy, although to a lesser extent than pre-onset treatment.

**Conclusions:**

Caspase-1 inhibition effectively ameliorates both systemic and renal manifestations of SLE in MRL-*Fas^lpr^* mice, primarily through suppression of IL-18-mediated inflammation. This study identifies caspase-1 as a promising therapeutic target for SLE and LN, warranting further investigation in clinical trials.

**WHAT IS ALREADY KNOWN ON THIS TOPIC**

- Inflammasome activation and increased caspase-1 activity are involved in the pathogenesis of systemic lupus erythematosus (SLE) and lupus nephritis (LN), primarily through the maturation of the proinflammatory cytokine interleukin (IL)-18. While IL-18 is recognised as a key mediator of disease severity, the therapeutic potential of targeting caspase-1 pharmacologically had not been explored in lupus models.

**WHAT THIS STUDY ADDS**

- This study shows that selective caspase-1 inhibition in Murphy Roths large (MRL)-Faslpr mice markedly reduces systemic autoimmunity and kidney damage. The therapeutic effect is associated with suppression of IL-18 activity, decreased cytokine production, and reduced immune cell infiltration and activation.

**HOW THIS STUDY MIGHT AFFECT RESEARCH, PRACTICE OR POLICY**

- The findings support caspase-1 as a relevant and specific therapeutic target in SLE and LN. This may guide future research toward IL-18-centred therapeutic strategies and promote the development of caspase-1 inhibitors as potential disease-modifying treatments in autoimmune disorders.

## Introduction

 Systemic lupus erythematosus (SLE) is an autoimmune disease characterised by a dysregulated immune system, autoantibody production, deposition of immune complexes and damage to multiple organ systems.^1^ Renal involvement, so-called lupus nephritis (LN), is a severe manifestation that affects up to 50% of SLE patients and can lead to renal failure and an increased morbidity and mortality.^2 3^ MRL-*Fas^lpr^* mice serve as a well-established model of human SLE as they spontaneously develop a SLE-like phenotype at an age of 3 months including lymphoproliferation, autoantibody production and glomerulonephritis.^4^ A central mediator of SLE pathogenesis is interleukin (IL)-18, a proinflammatory cytokine from the IL-1 family, that promotes type 1 T helper (Th1) response, interferon (IFN)-γ production and renal inflammation, as evidenced by its elevated serum and glomerular expression in both human LN patients and murine lupus models.^5^,^10^ Various studies showed a correlation of IL-18 with disease activity or serological parameters.^911^,^13^ MRL-*Fas^lpr^* mice exhibit hyper-responsiveness to IL-18 due to constitutive overexpression of the IL-18 receptor β-chain (IL-18Rβ), amplifying IFN-γ-driven lymphoproliferation and renal injury.^14^ IL-18 receptor α (IL-18Rα)-deficient MRL-*Fas^lpr^* mice show attenuated nephritis, reduced autoantibody titres and prolonged survival.^15^ In contrast to IL-18, the role of IL-1β—another caspase-1-activated IL-1 family member—in SLE remains ambiguous. Although IL-1β antagonists like anakinra and canakinumab approved for rheumatoid arthritis and cryopyrin-associated periodic syndromes, the effect of IL-1 blockade in SLE is less clear.^16^ While IL-1β deficiency in mice injected with monoclonal anti-DNA antibody resulted in an ameliorated disease,^17^ human studies report inconsistent associations between IL-1β levels and SLE disease activity, with some detecting weak correlations^18^,^20^ and others finding no significance.^9 21^

IL-18 and IL-1β share a common activation mechanism, as both are cleaved from inactive precursors by caspase-1, a key enzyme in inflammasome signalling, to become biologically active. Inflammasome activation and subsequent caspase-1-dependent IL-18 maturation have been implicated in SLE pathogenesis, with studies demonstrating heightened caspase-1 activity in peripheral blood monocytes of SLE patients and an increased expression of inflammasome components, including NLR family pyrin domain containing 3 (NLRP3) and caspase-1 in LN biopsies.^22 23^ Caspase-1 inhibition could inhibit the activation of IL-18 and alleviate LN. Carbobenzoxy-valyl-alanyl-aspartyl-(β-o-methyl)-fluoromethylketone (ZVAD-fmk) is a pancaspase inhibitor that inhibits a broad spectrum of caspases, in particular caspase-1, caspase-3 and caspase-11 with high affinity in the murine model.^24^,^28^ It ameliorates renal disease in IFN-γ-transgenic lupus models by reducing apoptosis-mediated nucleosome release and immune complex deposition.^29^ Combination therapy of mycophenolate mofetil, tacrolimus and steroids suppressed NLRP3 and caspase-1 activation in vitro and in vivo in MRL-*Fas^lpr^* mice and patients associated with reduced disease activity.^30^ Caspase-1 knockout mice with pristane-induced lupus showed an ameliorated disease.^31 32^ The development of pralnacasan, a selective oral caspase-1 inhibitor, was evaluated to target inflammasome-driven pathologies in various autoimmune diseases and demonstrated its efficacy in reducing IL-1β and IL-18 maturation and attenuating inflammation in various murine models.^33^,^36^ A phase II trial with rheumatoid arthritis patients was discontinued after a long-term animal toxicology study revealed liver abnormalities at high doses.^34^ A wide range of diseases, including inflammatory, neurological, cancer and metabolic disorders, are linked to the dysregulation of caspase-mediated cell death and inflammation and caspase inhibitors are evaluated with the aim of therapeutic application.^34^ However, due to limited efficacy or toxic side effects, only a few caspase inhibitors have progressed to clinical trials, and none has yet proven successful for clinical use.^34^ Caspase-1 is not only responsible for the activation of IL-1β and IL-18, but also triggers pyroptosis, an inflammatory form of programmed cell death that leads to cell lysis and release of cytosolic contents into the extracellular space thus seems well suited for SLE treatment. We therefore tested the effect of inhibition in lupus mice to provide evidence for further pursuit in the development of new drugs associated with this approach. While genetic deletion^31 32^ and pancaspase inhibition have previously been shown to ameliorate lupus in murine models,^29^ our study extends this work by investigating efficacy with a selective caspase-1 inhibitor. To our knowledge, no pharmacological caspase-1 inhibition was previously investigated in a murine SLE model. This study aims to clarify: (1) The therapeutic potential of caspase inhibition (using ZVAD-fmk and pralnacasan) in MRL-*Fas^lpr^* mice and check whether IL-18 and/or IL-1β have influence on the pathogenesis. (2) Whether intervention post-disease onset can halt or reverse LN. (3) The specificity of caspase-1 inhibitors for their target, testing if they influence other caspases and interleukins. (4) Broader immunomodulatory effects, including cytokine profiles and immune cell activation.

## Material and methods

### Mice

MRL-*Fas^lpr^* (Strain No. 000485) mice were purchased from Jackson Lab. Mice were bred and held in the translational animal research centre (TARC), University Medical Center Mainz. Female mice were used in all experiments. In a preliminary trial, 4-month-old mice were treated with ZVAD-fmk or saline with daily subcutaneous injection for 28 days. The treatment group received 10 mg/kg ZVAD-fmk (Alexis Biochemicals, CH) dissolved in Dimethyl sulfoxide (DMSO) and diluted to 1 mg/mL in 0.15 M saline. Control animals were injected with the corresponding volume of saline-DMSO diluent.

In a therapeutic approach, MRL-*Fas^lpr^* mice at 4 months of age with advanced LN received oral caspase-1 inhibitor (pralnacase by administering food–drug mixtures ad libitum at concentrations of 4200 ppm (mg/kg food)^33^) or control chow for 4 weeks. In a prophylactic approach, MRL-*Fas^lpr^* mice at 2.5 months of age received the therapy or control chow for 3 months.

To investigate the role of IL-1β, MRL-*Fas^lpr^* mice at 4 months of age were treated subcutaneously with IL-1 receptor antagonist (IL-1RA; Amgen) at a concentration of 10 mg/kg weight or Phosphate-buffered saline (PBS) in the control group.

Sample sizes were based on previous studies using the MRL-*Fas^lpr^* model and aimed to detect biologically relevant differences while minimising animal use. No formal statistical power calculation was performed. Animals were randomly allocated to treatment groups using computer-generated random numbers before treatment initiation. Due to technical limitations or insufficient sample material, group sizes varied between experiments, as detailed in the figure legends. No animals were excluded based on treatment response. Where possible, data analysis (eg, histological scoring) was performed blinded to the group allocation. However, feeding with different diets and administration of medications via subcutaneous injection were not blinded.

After treatment, urine and tissue samples were taken. The main outcome was disease activity, assessed by composite phenotype, kidney damage (pathology scores, apoptosis, proteinuria, immune cell infiltration) and systemic inflammation parameters.

Use of mice in this study was reviewed and approved by the Standing Committee on Animals (Protocol #G-14-1-054), in adherence to standards set in the Guide for the Care and Use of Laboratory Animals.

### Renal pathology

Kidneys were fixed in 10% neutral-buffered formalin for 24 hours, after which paraffin sections (4 µm) were stained with periodic acid-Schiff’s reagent. Kidney pathology was evaluated using a previously described method.^37^ Briefly, the assessment of glomerular pathology involved scoring each glomerulus on a semiquantitative scale: 0=normal (35–40 cells/glomerular cross-sections (gcs)); 1=mild (glomeruli with few lesions showing slight proliferative changes, mild hypercellularity (41–50 cells/gcs)); 2=moderate (glomeruli with moderate hypercellularity (51–60 cells/gcs), including segmental and/or diffuse proliferative changes, hyalinosis); 3=severe (glomeruli with segmental or global sclerosis and/or severe hypercellularity (80–60 cells/gcs), necrosis, crescent formation); 4=most severe (glomeruli with extensive global sclerosis and/or most severe hypercellularity (>80 cells/gcs), widespread necrosis, multiple crescent formation and severe fibrosis). We scored all glomeruli of the section. Interstitial/tubular pathology was assessed semiquantitatively on a scale of 0–3 in 10 randomly selected high-power fields (hpf). The largest and average number of infiltrates and damaged tubules were determined, and the grading system was adjusted accordingly: 0=normal, 1=mild, 2=moderate, 3=severe. Perivascular cell accumulation was evaluated semiquantitatively by scoring the number of cell layers surrounding the majority of vessel walls (score: 0=none, 1 = <5 cell layers, 2=5–10 cell layers, 3 = >10 cell layers). All histological evaluations were performed blinded and independently by two investigators, and the final score for each parameter was calculated as the mean of their assessments.

### Immunostaining

Kidney tissue was processed and stained for the presence of F4/80, CD4, CD8 and B220 as previously described.^38^ Antibodies are listed in online supplemental table 1. We determined the number of positive cells in 10 randomly selected hpf.

### RNAse Protection Assay (RPA)

The RNAse Protection Assay (RPA) allows us to study the relative mRNA levels of hundreds of genes of cytokines and caspases and especially their differential expression in response to particular physiological or pathological conditions simultaneously. The RPA kit from PharMingen with the template sets mouse anti–Apo-1 (mApo-1) for caspase detection (caspase-1, caspase-2, caspase-3 to caspase-6, caspase-7 to caspase-8, caspase-11 to caspase-12) and mouse cytokines/chemokines template set (mCK-3b) for detection of mRNA expression of cytokines (IL-18, IL-1β, IFN-γ, IL-12A, IL-12B, IL-10, IL-1 α) was used. First, total RNA from tissue was isolated from kidney and spleen tissue using Quiagen RNeasy Mini Kit (Qiagen, Hilden) according to the manufacturer’s instructions. The RNA concentration was quantified by optical density (OD) measurement of the absorbance at 260 nm and 280 nm in a spectrometer (Nanodrop 2000). RNA probes were radioactively labelled with phosphor (P)^32^ via in vitro transcription and subsequently hybridised with isolated RNA samples. After RNase digestion of the non-hybridised RNA, the samples were applied to a 5% acrylamide gel. The dried gel was developed on film. The housekeeping genes ribosomal protein L32 (L32) and glyceraldehyde-3-phosphate dehydrogenase (GAPDH) were used as controls. The band intensity of the individual probes was evaluated densitometrically using the National Institutes of Health (NIH) image 1.62 computer software and placed in relation to the band density of the respective housekeeping genes. The ratio was stated as a ‘densitometric unit’.

### Caspase substrate assay

For activation, the caspases pro-proteins are cleaved to subunits. The substrate of this assay corresponds to one of the cleavage sites of the caspase (Z-YVAD-AFC caspase-1 fluorogenic substrate (AFC); Ac-DEVD-AFC for caspase-3; Ac-IETD-AFC for caspase-6 and caspase-8; Ac-LEHD-AFC for caspase-9, Biomol International LP). After cleavage of the substrate by respective caspases, the reaction was visualised and quantified by a fluorescence microtiter plate reader. When excitation wavelength is 400 nm, free AFC emits a yellow-green light. Tissue samples of kidney and spleen (~0.01 g) were crushed and lysed. Protein amounts were determined. Protein samples were lysed in Cell Lysis Buffer, after 10 min incubation on ice, 50 µL dithiothreitol (DTT) 10 mM was added. To quantify the caspase activity, 5 µL of 1 mM respective substrate solution was given to each sample. After 90 min incubation in the dark at 37°C, the fluorescence activity (400 nm excitation, 505 nm emission) was determined.

### FACS

Fluorescence-activated cell sorting (FACS) analysis was done as described before.^39^ In brief: renal tissue was digested with collagenase IV to achieve a single cell solution. After lysis of red blood cells, kidney cells were resuspended in FACS buffer (1× PBS/3% fetal bovine serum (FBS)). Antibodies and staining kits are listed in online supplemental table 1.

### Proteome profiler

Renal tissue lysates were used for cytokine and chemokine quantification using the Proteome Profiler Mouse Cytokine Array Kit, Panel A (by R&D). Analysis was done according to the manufacturer’s instructions.

### ELISA

To quantify the levels of IL-18, IL-12, IL-1β, TNF-α and IFN-γ in sera, we performed ELISA using the following kits: OptEIA Mouse kit, IL-18 Cat. No. 2694KI, IL-1β Cat. No. 550603, IL-12 Cat. No. 555256, TNF-α Cat. No. 558874 and IFN-γ Cat. No. 555138 (PharMingen BD Bioscience). ELISAs were performed according to the manufacturer’s instructions.

### Proteinuria and urea

Urine protein levels were assessed semiquantitatively using Multistix (BAYER Vital GmbH, Germany) (0=none; 0,5=30 mg/dL; 1=100 mg/dL; 2=300 mg/dL; 3 = >2000 mg/dL). The concentrations of urea in sera were measured by the central laboratory of the Johannes Gutenberg-University of Mainz (UREA/blood urea nitrogen (BUN) kinetic UV-Test, Cat. No. 1982486001V8, Roche, F).

### Anti-double-stranded DNA antibodies

Anti-double-stranded DNA antibodies were analysed using a commercially available ELISA kit (Pharmacia Diagnostic, Art. No. 14148/14196). Analysis of serum samples (dilution of 1:200) was done according to the manufacturer’s instructions.

### Skin lesions

Skin lesions were scored as described before^39^ using a score of 0–3 (0=none; 1=mild (snout and ears); 2=moderate, <2 cm (snout, ears, and intrascapular); 3=severe, 2–4 cm (snout, ears and intrascapular); and 4=very severe, >4 cm (snout, ears, and intrascapular)).

### TUNEL staining

Apoptotic cells were identified using enzymatic in situ labelling of apoptosis-induced DNA strand breaks (terminal deoxynucleotidyl transferase dUTP nick end labelling (TUNEL) method). Frozen sections were fixed in 4% paraformaldehyde in PBS, then permeabilised with 0.1% Triton X-100 in 0.1% sodium citrate for 2 min on ice. Labelling with TUNEL reaction mixture containing terminal deoxynucleotidyl transferase and fluorescein-labelled nucleotides (Boehringer Mannheim) occurred for 60 min at 37°C. Subsequently, incorporated nucleotides were labelled with sheep anti-fluorescein Fab labelled with horseradish peroxidase (1/5 dilution; Boehringer Mannheim) for 30 min at 37°C. 3,3′-diaminobenzidine (DAB) (Vector Laboratories) was used to stain apoptotic cells. Tissue sections were counterstained with methyl green/Alcian blue. The number of positive cells in at least 5 randomly selected hpf was scored by two blinded observers. Additionally, TUNEL-positive cells were examined under light microscopy for morphological characteristics of apoptosis, such as condensed and fragmented nuclei.

### Statistics

Statistical analyses were performed using GraphPad Prism 10.4.1 software. Normality was tested using the Anderson-Darling and Shapiro-Wilk tests, and the tests used are indicated in the figure legends.

## Results

### Pharmacological caspase-1 inhibition ameliorates disease phenotype in MRL-Fas^lpr^ mice

**Figure 1:**
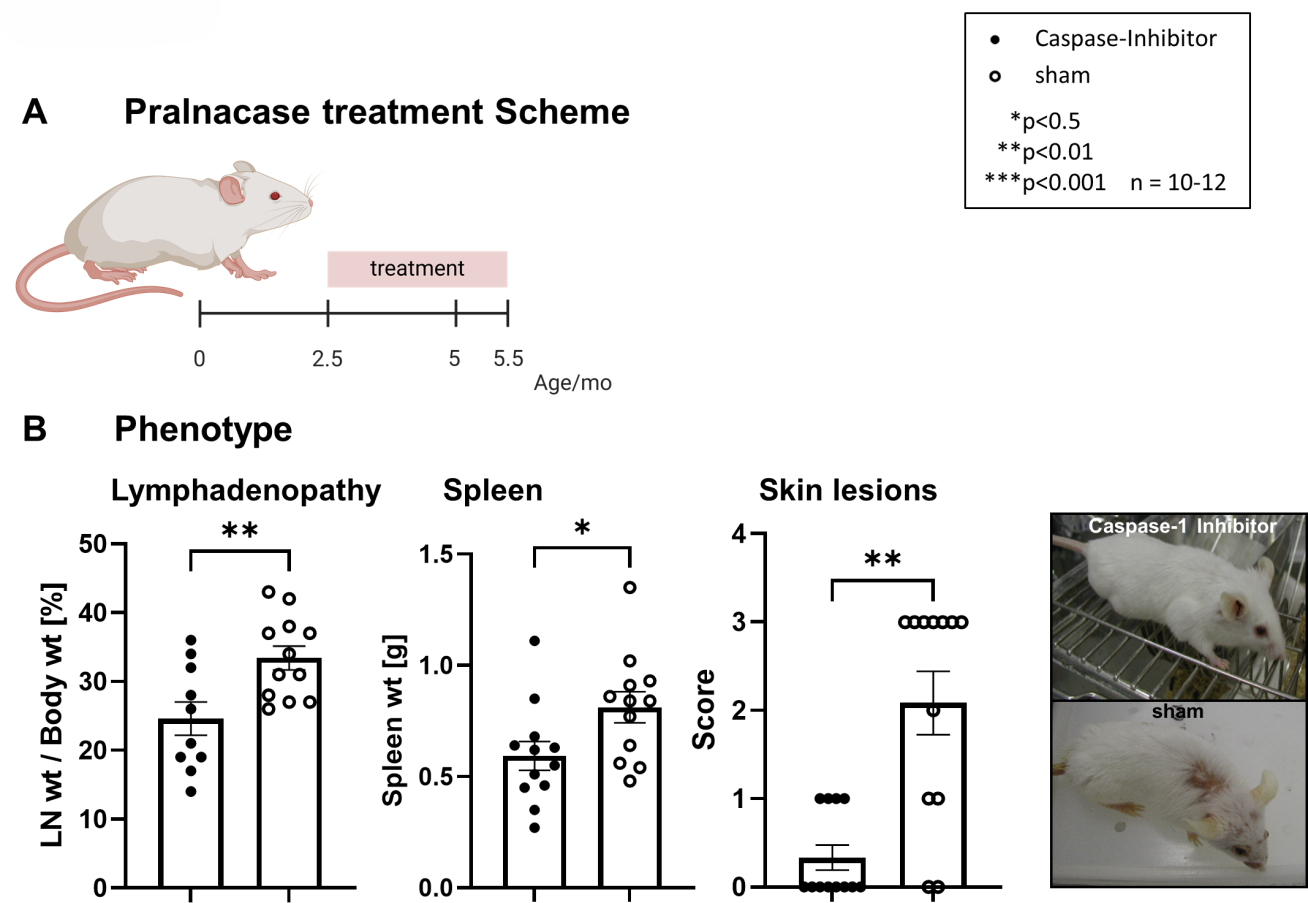
Pralnacase treatment ameliorates disease in mice. (**A**) Schematic representation of the pralnacase treatment regimen. Mice were treated starting at 2.5 months of age until 5.5 months of age (indicated by the red bar). (**B**) Phenotypic analysis of lymphadenopathy, spleen weight and skin. Lymphadenopathy was assessed as lymph node weight (LN wt) relative to body weight (%) and analysed using a two-tailed unpaired t-test (**p<0.01). Spleen weight (**g**) was also analysed using a t-test (*p<0.05). Skin lesion severity was scored on a scale of 0–4 and analysed using the Mann-Whitney U test (**p<0.01). Representative images of mice treated with the caspase-1 inhibitor or sham treatment are shown on the right, illustrating reduced skin lesions in treated animals. Data are presented as mean±SEM, with n=10–12 animals per group.

To assess the therapeutic potential of targeting IL-18 via caspase-1 inhibition, we treated MRL-*Fas^lpr^* mice from 2.5 to 5.5 months of age with a selective caspase-1 inhibitor (figure 1A), which prevents the conversion of the inactive pro-IL-18 into the mature IL-18. Early intervention (pre-disease onset) significantly attenuated systemic manifestations, including lymphadenopathy, splenomegaly and skin lesions when compared with sham-treated MRL-*Fas^lpr^* mice (figure 1B). Body weight did not differ significantly (data not shown).

**Figure 2:**
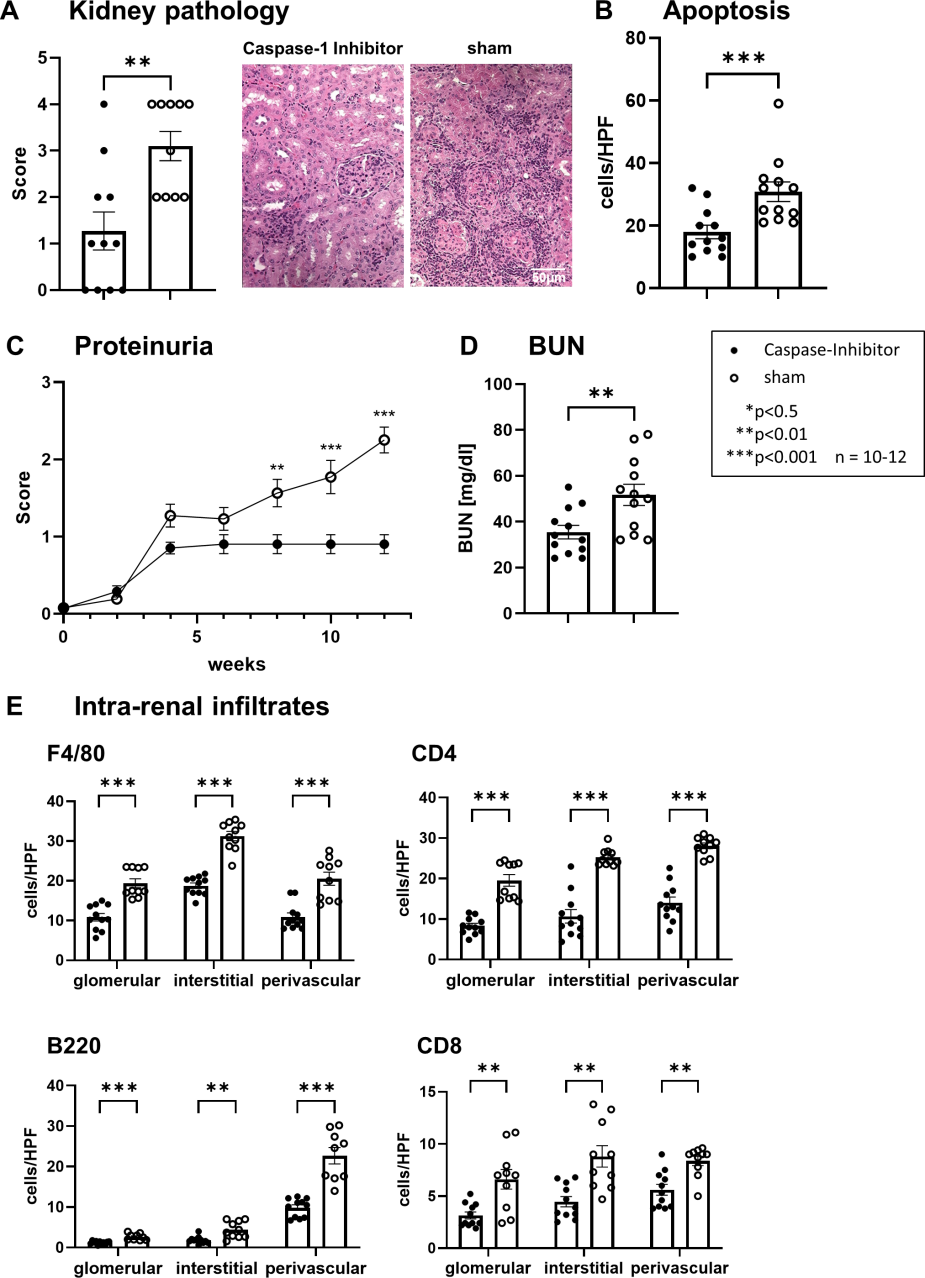
Pralnacase treatment reduces kidney pathology, apoptosis and inflammation. (**A**) Kidney pathology scores. Pathology was assessed on a semiquantitative scale (0–4), with representative PAS-stained kidney sections shown on the right. Statistical analysis was performed using the Mann-Whitney U test (**p<0.01). (**B**) Quantification of apoptotic cells in kidney sections, determined by terminal deoxynucleotidyl transferase dUTP nick end labelling (TUNEL) staining. Data are expressed as the number of TUNEL-positive cells per high-power field (hpf). Statistical analysis was performed using the Mann-Whitney U test (***p<0.001). (**C**) Proteinuria scores over time. Proteinuria was scored weekly for 12 weeks. Statistical analysis was performed using two-way analysis of variance (ANOVA) with Šídák’s multiple comparisons test (**p<0.01, ***p<0.001). (**D**) Blood urea nitrogen (BUN) levels. Statistical analysis was performed using a two-tailed unpaired t-test (**p<0.01). (**E**) Quantification of intra-renal immune cell infiltrates, including F4/80^+^ macrophages, CD4^+^ T cells, B220^+^ B cells and CD8^+^ T cells, in glomerular, interstitial and perivascular regions. Data are expressed as the number of positive cells per hpf. Statistical analysis was performed using a two-tailed unpaired t-test with Šídák’s multiple comparisons test (**p<0.01, ***p<0.001). All data are presented as mean±SEM, with n=10–12 animals per group.

**Figure 3:**
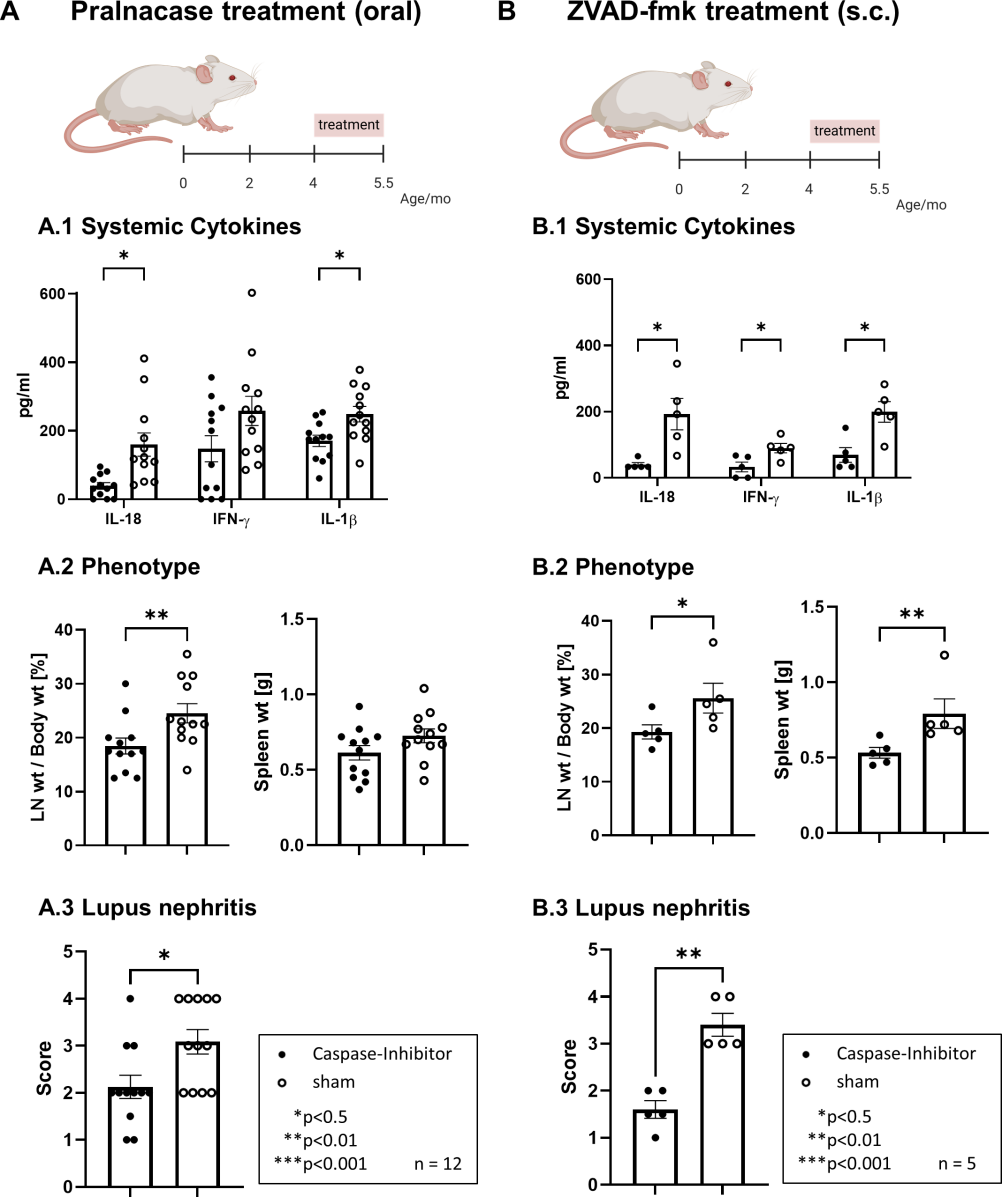
Pralnacase and Carbobenzoxy-valyl-alanyl-aspartyl-(β-o-methyl)-fluoromethylketone (ZVAD-fmk) treatments reduce systemic inflammation and disease severity. (**A**) Pralnacase treatment in a therapeutic approach (oral) and (**B**) ZVAD-fmk treatment (subcutaneous): A/B.1 Systemic cytokines: levels of IL-18, interferon (IFN)-γ and IL-1β in serum were measured by ELISA. Statistical analysis was performed using multiple Mann-Whitney U tests with Holm-Šídák correction (*p<0.05). A/B.2 Phenotype: lymphadenopathy (LN weight as % of body weight) and spleen weight (**g**) were assessed. Statistical analysis was performed using the Mann-Whitney U test (*p<0.05, **p<0.01). A/B.3 Lupus nephritis: kidney pathology scores were determined on a semiquantitative scale (0–4). Statistical analysis was performed using the Mann-Whitney U test (*p<0.05). All data are presented as mean±SEM, with n=12 animals per group for pralnacase treatment and n=5 animals per group for ZVAD-fmk treatment.

The treatment also alleviates LN (figure 2). Treated mice exhibited attenuated glomerular and tubular pathology (figure 2A) and apoptosis (TUNEL staining; figure 2B). An improved renal function determined by proteinuria and blood urea nitrogen was observed (BUN; figure 2C/D). Furthermore, the number of infiltrating macrophages (F4/80^+^), B cells (B220^+^) and T cells (CD4^+^/CD8^+^) was significantly reduced as shown by immunohistochemistry (figure 2E). Therapeutic intervention post-disease onset (4.0–5.5 months) also mitigated renal and systemic pathology, although with reduced efficacy compared with pre-onset treatment (figure 3A). Subcutaneous administration of the pancaspase inhibitor ZVAD-fmk with inhibition of various caspases (in particular caspase-1, caspase-3 and caspase-11) led to a comparable therapeutic effect (figure 3B), which indicates the central importance of caspase-1.

Taken together, caspase inhibition suppressed disease progression in MRL-*Fas^lpr^* mice with caspase-1 as central mediator.

### Caspase-1 inhibitor exhibits target specificity

**Figure 4:**
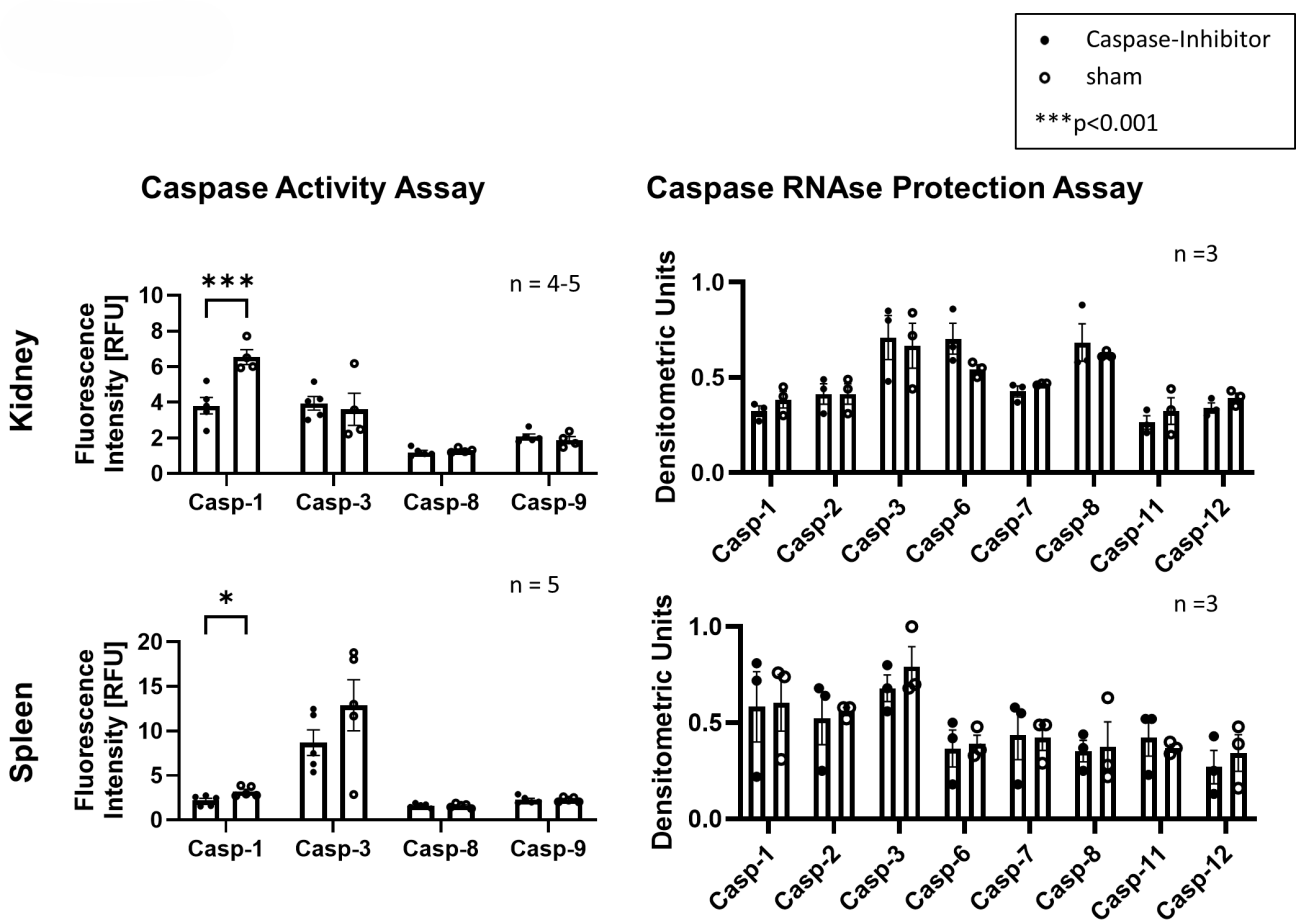
Pralnacase treatment reduces caspase-1 activity in kidney and spleen selectively, without affecting RNA expression levels. Caspase activity assay: fluorescence intensity (RFU) was measured for caspase-1, caspase-3, caspase-8 and caspase-9 in kidney (top left) and spleen (bottom left). Statistical analysis was performed using a two-tailed t-test with Holm-Šídák correction (*p<0.05, ***p<0.001). Caspase RNAse protection assay: densitometric units of caspase mRNA expression (caspase-1, caspase-3, caspase-7 to caspase-8, caspase-9 to caspase-11, and caspase-12) were quantified in kidney (top right) and spleen (bottom right) tissues. Statistical analysis was performed using a two-tailed t-test with Holm-Šídák correction (*p<0.05). All data are presented as mean±SEM, with n=5 for kidney and n=4–5 for spleen samples.

To confirm the specificity of caspase-1 inhibition of Pralnacase, we analysed enzymatic activity and RNA expression of caspases in kidney and spleen lysates. Using a caspase-enzyme assay, we assessed caspase-1 inhibitor affects only the activity of caspase-1 while activities of caspase-3, caspase-8 and caspase-9 remained unchanged in kidney and spleen (figure 4). Furthermore, RNA expression levels of caspase-1, caspase-3, caspase-8 and caspase-9 were unaffected by treatment (figure 4), confirming the selectivity for caspase-1 enzymatic activity without transcriptional off-target effects.

### IL-18, but not IL-1*β,* drives disease in MRL-Fas^lpr^ mice

**Figure 5:**
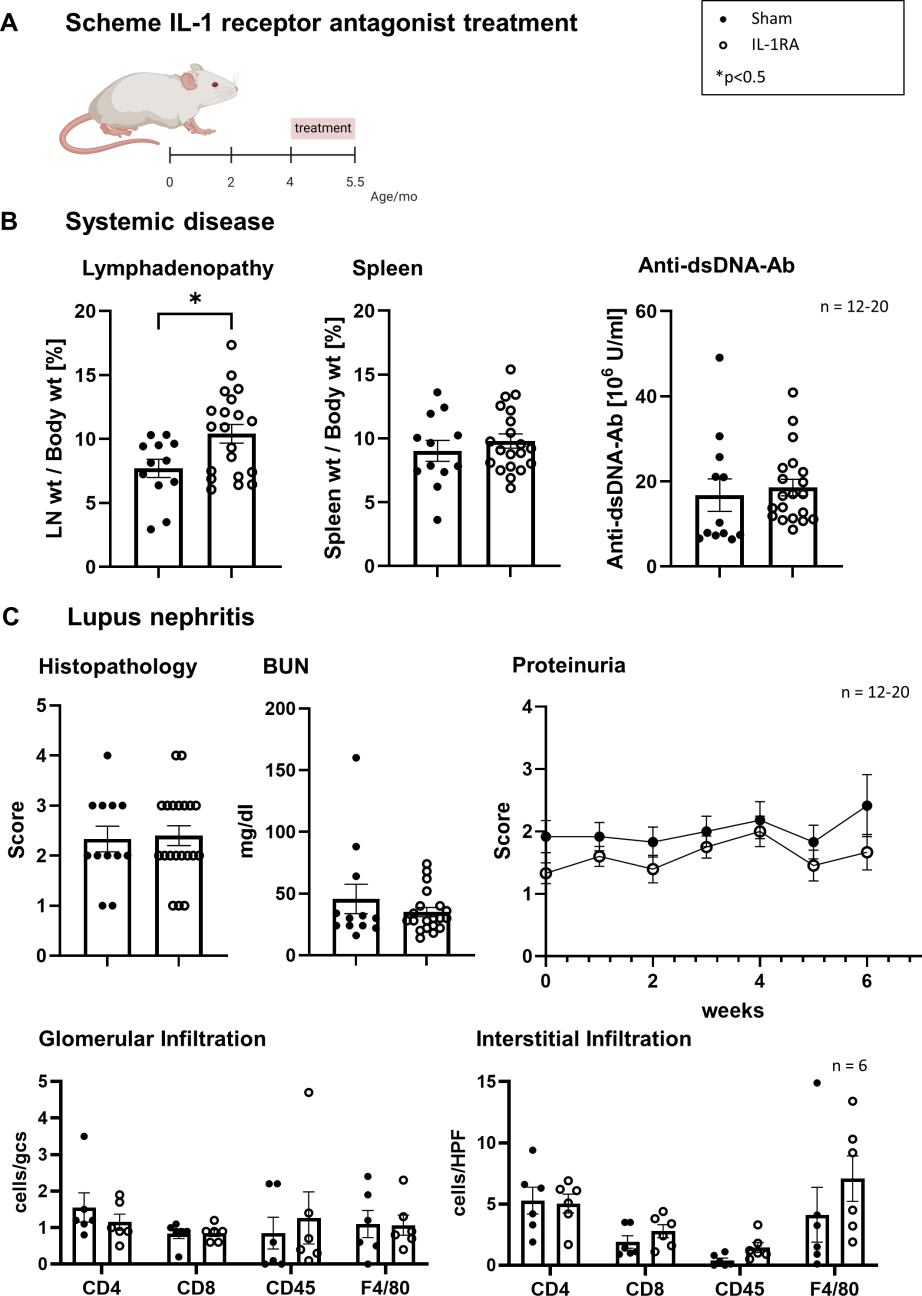
IL-1 receptor antagonist (IL-1RA) treatment fails to reduce systemic disease and LN. (**A**) Schematic representation of the IL-1 receptor antagonist (IL-1RA) treatment regimen. Mice were treated from 4 to 5.5 months of age (indicated by the red bar). (**B**) Lymphadenopathy was assessed as lymph node weight (LN wt) relative to body weight (%), spleen weight was measured relative to body weight (%), and serum anti-dsDNA antibody levels were quantified. Statistical analysis was performed using an unpaired t-test (*p<0.05). (**C**) Lupus nephritis: kidney histopathology scores were determined on a semiquantitative scale (0–4). Glomerular and interstitial immune cell infiltration (CD4^+^ T cells, CD8^+^ T cells, CD45^+^ leukocytes and F4/80^+^ macrophages) was quantified as the number of positive cells per high-power field (hpf). Blood urea nitrogen (BUN) levels were measured in mg/dL, and proteinuria was scored weekly over 6 weeks. Statistical analysis for all parameters was performed using an unpaired t-test. All data are presented as mean±SEM, with n=6–20 animals per group, as indicated in each panel.

Since caspase-1 activates both IL-18 and IL-1β, we treated mice with an IL-1 receptor antagonist (IL-1RA) to dissect their contributions. Although we could detect an improved lymphadenopathy, splenomegaly and levels of anti-dsDNA antibodies were not affected in IL-1RA treated compared with sham-treated mice (figure 5B). Furthermore, the renal function and the extent of the renal pathology as well as the number and phenotype of infiltrating intra-renal leukocytes were not affected by IL-1RA treatment (figure 5C). Moreover, the number of apoptotic tubular epithelial cells (TEC) and proliferating cells did not show any significant difference (data not shown).

Therefore, we assume that the amelioration of the disease observed in MRL-*Fas^lpr^* mice by caspase-1 inhibition was primarily mediated by the suppression of active IL-18 and less prominently by the reduction of IL-1β.

### Caspase-1 inhibition by pralnacase reduces proinflammatory cytokine production

**Figure 6:**
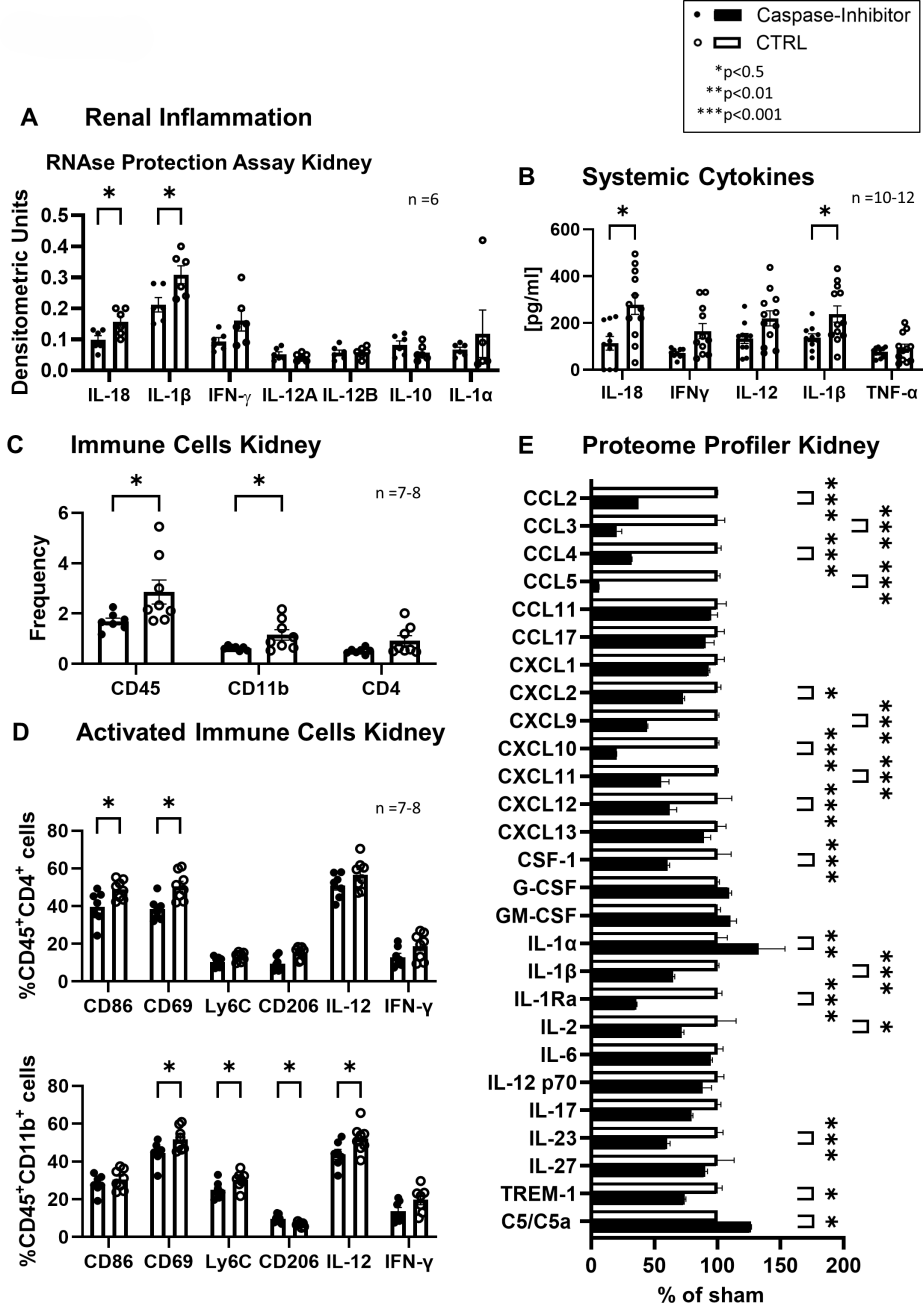
Caspase inhibition reduced renal inflammation and systemic cytokine levels. Less immune cell infiltration and activation were observed. (**A**) Renal inflammation: mRNA expression of inflammatory cytokines in kidney tissue was quantified using an RNAse protection assay. Statistical analysis was performed using multiple t-tests without correction for multiple testing due to the exploratory nature of the analysis (p<0.05). (**B**) Systemic cytokines were measured by ELISA. Statistical analysis was performed using a t-test with Holm-Šídák correction (p<0.05, p<0.01). (**C**) Immune cell populations in the kidney: frequencies of CD45^+^, CD11b^+^ and CD4^+^ immune cells were determined by flow cytometry. Statistical analysis was performed using a two-tailed unpaired t-test (p<0.05). (**D**) Activated immune cells in the kidney: Frequencies of activated CD45^+^CD4^+^ T cells and CD45^+^CD11b^+^ myeloid cells expressing activation markers were quantified by flow cytometry. Statistical analysis was performed using a two-tailed unpaired t-test (p<0.05). (**E**) Proteome profiler for kidney tissue was measured as a percentage of sham-treated controls. Statistical analysis was performed using a t-test with Holm-Šídák correction (p<0.05, p<0.01). All data are presented as mean±SEM, with n=6–12 animals per group as indicated in each panel.

To examine the mechanism of action of the caspase-1 inhibition, we measured systemic cytokine levels. We detected decreased levels of IL-18 and IL-1β as well as trends toward reduced IFN-γ and IL-12 following caspase-1-inhibitor treatment compared with sham-treated mice (figure 6B). In addition, a therapeutic treatment past the onset of disease with a caspase-1 inhibitor resulted in decreased levels of IL-18, IL-1β and IFN-γ tendentially (figure 3A1). These findings go along with reduced mRNA detection for the corresponding cytokines (figure 6A).

### Attenuated immune cell activation

Another mechanism could be reduced immune cell activation. Flow cytometry demonstrated reduced CD45^+^ leukocyte infiltration and CD11b^+^ macrophage counts in pralnacase-treated kidneys, with trends toward lower CD4^+^ T cell counts (figure 6C). Among CD4^+^ T cells, activation markers CD69 and CD86 were significantly downregulated (figure 6D). In addition, CD11b^+^ macrophages showed reduced expression of the activation markers CD69, Ly6C, CD206 and IL-12 (figure 6D), suggesting dampened proinflammatory polarisation. Additionally, IFN-γ production in both T cells and macrophages showed a trend toward reduction, further highlighting diminished adaptive-immune activation. Thus, treatment with caspase-1 inhibitors appears to attenuate the activation of T cells and macrophages.

### Attenuated immune cell recruitment

Caspase-1 inhibition also disrupted leucocyte recruitment to renal tissue. Proteome profiling identified reduced expression of chemokines critical for immune cell migration (figure 6E). Reduced expression of C-C motif chemokine ligand (CCL)2 (monocyte recruitment), CCL3/4/5 (granulocyte recruitment), C-X-C motif chemokine ligand (CXCL)9/10/11 (Th1 cell recruitment) and colony-stimulating factor 1 (CSF-1) (differentiation and survival factor of macrophages) was observed (figure 6E). Additionally, the proteome analysis confirms the reduced expression of interleukins such as IL-1 superfamily, IL-2 and IL-23.

Taken together, these findings indicate that inhibition of caspase-1 led to a decrease in leucocyte migration factors, leucocyte infiltration and leucocyte activation. Treatment also resulted in a reduction of proinflammatory cytokine release, collectively contributing to a mitigation of kidney damage.

## Discussion

The findings of this study show a central role of caspase-1 in driving LN and systemic autoimmunity in MRL-*Fas^lpr^* mice, likely mediated by IL-18 activation. This is supported by evidence highlighting caspase-1 as a key mediator of inflammasome dysregulation in SLE.^8 31^ Elevated caspase-1 activity as a result of increased inflammasome activation in monocytes was observed in SLE patients and correlated with disease severity.^22 40^ It has already been described inflammasome inhibition by targeting NLRP3 or cyclic GMP-AMP synthase-stimulator of interferon genes (cGAS-STING) pathway had the potential to downregulate caspase-1 activity induced by SLE serum in human monocytes.^40^ Further inhibition of NLRP3 attenuated disease in MRL-*Fas^lpr^* mice.^41^ A combination therapy (mycophenolate mofetil, tacrolimus and steroids) suppresses the activation of NLRP3 and caspase-1 associated with reduced gasdermin D in cultured podocytes and in humans slows down disease progression through inhibition of caspase-1/gasdermin D (GSDMD)-mediated pyroptosis.^30^ While caspase-1 inhibition by genetic deletion in inducible lupus models has already been shown to protect against autoantibody formation, type I interferon responses, vascular dysfunction and immune complex glomerulonephritis,^32^ we demonstrate in our work that selective pharmacologic caspase-1 inhibition is effective in a spontaneous lupus model, ameliorating systemic disease and lupus nephritis. Since caspase-1 in particular is of great importance in various autoimmune processes and at the same time its inhibition suppressed central interleukins in the pathogenesis of LN, caspase-1 appears to be an ideal target.^34^ Compared with pancaspase inhibition, less side effects can be assumed with specific inhibition of caspase-1. Hence, essential apoptotic pathways are preserved while mitigating pyroptosis-driven inflammation. We have demonstrated pralnacase selectively inhibits caspase-1 (figure 4). Furthermore, caspase-1 inhibition is not inferior to the broader pancaspase inhibition in our experiment (figure 3).

While caspase-1 inhibition affects both biologically active IL-18 and IL-1β, our data align with prior clinical studies establishing IL-18 as the principal driver of LN.^6^,^8^ The weak correlation between IL-1β and SLE activity in humans,^9^ as well as the failure of IL-1 receptor antagonism to ameliorate LN in our model, reinforces the concept that IL-18, more than IL-1β, mediates LN as a central interleukin in SLE. This difference may result from a stronger capacity of IL-18 than IL-1β to synergise with IL-12 to induce IFN-γ production, affecting the function of a variety of immune cells (eg, differentiation and activation of T cell, B cell, macrophage and dendritic cells).^42 43^ The reduction in IFN-γ and Th1-associated chemokines (CXCL9/10/11) in treated mice further supports this mechanism. Overall, we consider the following three major changes to be responsible for the attenuated renal pathology as a result of the pralnacase treatment: (1) lower expression of cytokines both systemically and in renal tissue, (2) reduced leucocyte infiltration in the kidney, accompanied by decreased expression of chemokines that promote leucocyte migration and (3) reduced activation of renal T cells and macrophages. In line with our observations, it was shown that caspase-1 inhibition could promote M2 polarisation of macrophages and suppress nuclear factor kappa B (NF-κB) activation.^44^

Caspase inhibition led to the expected reduction of IL-18 and IL-1β. Proteome profiler analyses further showed that CXCL9, CXCL10 and CXCL11 were also decreased, potentially mediated by reduced IL-18–induced IFN-γ release.^45 46^ In contrast, other cytokines important for the pathogenesis of lupus nephritis remained unchanged. This pattern may reflect selectivity of caspase inhibition, but could also depend on disease stage, as our analyses focused on advanced disease.

The discontinuation of pralnacasan development due to long-term hepatotoxicity at supratherapeutic doses in animals has raised concerns about caspase-1 inhibitors. However, our data showed significant benefits of caspase-1 inhibition in LN, such that these side effects could possibly be mitigated through refined dosing regimens or targeted delivery (eg, renal-targeted nanoparticles), while preserving efficacy. Furthermore, another caspase-1–specific inhibitor, belnacasan (VX-765), completed a phase II trial in patients with epilepsy showing safety and tolerability. Recent research demonstrated VX-765 ameliorates vascular inflammation and atherosclerosis in a murine model of atherosclerosis,^47^ suggesting another potential therapeutic benefit of caspase-1 inhibition for SLE patients, as cardiovascular events are a significant cause of death in this population. Notably, we were able to show that the treatment is also effective after the onset of the disease, underlining caspase-1 inhibition could be a relevant therapeutic approach.

Since antiphospholipid antibodies (aPL) can induce NLRP3 and caspase-1 activation, leading to inflammasome assembly and, via IL-1β and IL-18, promoting inflammation, oxidative stress and endothelial dysfunction,^48 49^ another potential application of caspase-1 inhibition is in antiphospholipid syndrome (APS), particularly in microvascular manifestations such as aPL-associated nephropathy. This hypothesis requires further investigation in dedicated experimental models to determine whether caspase-1 blockade can indeed mitigate aPL-driven vascular pathology.

While our study identifies caspase-1 as a promising therapeutic target, several questions remain unanswered. The contribution of caspase-1-independent IL-18 activation pathways (eg, proteinase-3) should be investigated. Although IL-1 receptor blockade showed no efficacy in our setting, contributions from IL-1β or non-canonical inflammasome pathways cannot be excluded. Future studies incorporating broader cytokine and chemokine profiling to assess IL-18 downstream signalling, as well as genetic or neutralising approaches targeting IL-18, may help to further clarify its role in these mechanisms. Additionally, the study is limited by the small cohort size and the lack of long-term efficacy and safety data. On the one hand, possible hepatotoxic effects; on the other hand, infective complications due to the dysregulated immune system in combination with IL1/IL-18 suppression. Furthermore, it remains to be seen whether inhibition of the activation of the NLRP3 inflammasome, as recently shown with mesenchymal stem cells in murine and human lupus, has a more favourable efficacy/side-effect profile.^50^ Moreover, monoclonal antibodies targeting IL-18, such as GSK-1070806 and camoteskimab, are currently under development for conditions like atopic dermatitis and Still’s disease, and may hold potential for therapeutic evaluation in SLE.

In summary, this study identified caspase-1 inhibition as a mechanistically based strategy to disrupt IL-18-driven inflammation, immune cell recruitment and pyroptosis in SLE. In the development of further caspase-1 inhibitors, SLE patients should be considered as a potential target group.

## Supplementary material

10.1136/rmdopen-2025-006194 online supplemental file 1

## Data Availability

Data are available upon reasonable request.
