# Supplementary material for: Treatment with caspase-1 inhibitor diminishes kidney disease in MRL-Faslpr mice and delays systemic illness
Source: RMD Open. 2025 Dec 23;11(4):e006194. doi: 10.1136/rmdopen-2025-006194 (PMC12730834; doi:10.1136/rmdopen-2025-006194)
Supplement: online supplemental file 1 [file rmdopen-11-4-s001.docx]

**Supplement**

| **Immunostaining** | | |  |  |
| --- | --- | --- | --- | --- |
|  | *F4/80* | *Rat-anti-mouse F4/80* | *CatNo: MCAP497* | *Serotec* |
|  | *CD4* | *Purified rat-anti-mouse CD4* | *CatNo: 553027* | *BD Pharmingen* |
|  | *CD8* | *Purified rat-anti-mouse CD8a* | *CatNo: 553027* | *BD Pharmingen* |
|  | *B220* | *Purified rat-anti-mouse CD45R/B220* | *CatNo: 553084* | *BD Pharmingen* |
|  |  |  |  |  |
| **FACS** | |  |  |  |
| *Antibodies* | |  |  |  |
|  | *CD4 FITC* | *FITC rat anti mouse CD4 CloneRM4-5* | *CatNo: 553047* | *BD Pharmigen* |
|  | *CD4 PerCP* | *PerCP Cy 5.5 conjugated anti-mouse CD4 Clone RM4-5* | *CatNo: 45-0042-82* | *eBioscience* |
|  | *CD11b APC* | *anti-mouse CD11b APC (M1/70)* | *CatNo: 17-0112-83* | *eBioscience* |
|  | *CD86 APC* | *conjugated anti-mouse CD86(B7-2) Clone GL1 APC* | *CatNo: 17-0862-81* | *eBioscience* |
|  | *CD69 PerCP* | *cPerCP Cy5.5 onjugated anti-mouse CD69(VEA) Clone H1.2F3* | *CatNo: 45-0691-82* | *eBioscience* |
|  | *Ly6C PE* | *rat anti mouse Ly-6c* | *CatNo: MCA2389PE* | *AbDSerotec* |
|  | *CD206 FITC* | *anti-mouse CD 206 MMR FITC Clone:C068C2* | *CatNo: 141704* | *Biolegend* |
|  | *IL-12 PE* | *anti-mouse IL12/IL23p40 (Clone C17.8)* | *CatNo: 12-7123-82* | *eBioscience* |
|  | *IFN-y PE* | *anti-mouse IFN-γ PE* | *CatNo: 554412* | *BD Pharmigen* |
| *Staining-Kit* | |  |  |  |
|  | *Th1/Th2/Th17 - Mouse Th1/Th2/Th17 Phenotyping Kit* | | *CatNo: 560758* | *BD Pharmigen* |
|  | *Th17/Treg - Mouse Th17/Treg Phenotyping Kit* | | *CatNo: 560767* | *BD Pharmigen* |

Table 1 Antibodies for Immunohistochemistry (IHC) and Fluorescence-Activated Cell Sorting (FACS) Analysis, and Staining Kits.
